# The Genetic Pathways Underlying Immunotherapy in Dilated Cardiomyopathy

**DOI:** 10.3389/fcvm.2021.613295

**Published:** 2021-04-14

**Authors:** Ayat Kadhi, Fathima Mohammed, Georges Nemer

**Affiliations:** ^1^Division of Genomics and Translational Biomedicine, College of Health and Life Sciences, Hamad Bin Khalifa University, Doha, Qatar; ^2^Department of Biochemistry and Molecular Genetics, Faculty of Medicine, American University of Beirut, Beirut, Lebanon

**Keywords:** dilated cardiomyopathy, immunomodilation, growth factors, precision medicine, immuno suppression

## Abstract

Heart failure (HF) is a global public health threat affecting 26 million individuals worldwide with an estimated prevalence increase of 46% by 2030. One of the main causes of HF and sudden death in children and adult is Dilated Cardiomyopathy (DCM). DCM is characterized by dilation and systolic dysfunction of one or both ventricles. It has an underlying genetic basis or can develop subsequent to various etiologies that cause myocardium inflammation (secondary causes). The morbidity and mortality rates of DCM remains high despite recent advancement to manage the disease. New insights have been dedicated to better understand the pathogenesis of DCM in respect to genetic and inflammatory basis by linking the two entities together. This cognizance in the field of cardiology might have an innovative approach to manage DCM through targeted treatment directed to the causative etiology. The following review summarizes the genetical and inflammatory causes underlying DCM and the pathways of the novel precision-medicine-based immunomodulatory strategies to salvage and prevent the associated heart failure linked to the disease.

## Introduction

Dilated Cardiomyopathy (DCM) is a common cause of Heart Failure (HF) and is the primary indication for heart transplantation. DCM is characterized by progressive dilatation and impaired contraction of one or both ventricles. The incidence of DCM has been estimated to be one case per 250 individuals, with a prevalence of >0.4% in the general population which accounts for 36% of HF cases (1, 2). DCM is responsible for about 10,000 deaths and 46,000 hospitalization each year in the United States making it amongst the top common causes of fatalities and a burden on health care system. These numbers might be an underestimation of the disease frequency because many affected patients have incomplete disease penetrance and expression (3, 4).

The World Health Organization (WHO) defines DCM as a serious cardiac disorder in which structural or functional abnormalities of myocardium that leads to cardiac malfunction, substantial morbidity and mortality, and complications such as heart failure and arrhythmias. The European Society of Cardiology (ESC) classifies cardiomyopathy into familial or non-familial (non-genetic) forms while the American Heart Association (AHA) committee classifies cardiomyopathies into three categories: “hereditary,” “mixed,” and “acquired” (5, 6). In respect to DCM, it is classified as a “mixed” disease and is best regarded as a complex trait with genetic and acquired/environmental components that promote cardiomyocyte injury or loss. DCM can occur due to a primary cause or in association with diverse range of conditions such as coronary artery disease, autoimmune disorders, inflammatory/infectious agents, chemotherapeutic drugs, toxins, alcohol excess or nutritional deficiencies. In about 50% of DCM cases, there is no known identifiable cause; this has been traditionally termed “idiopathic” DCM. Most of the times, the management of DCM aims at reducing symptoms, improving cardiac function, and prolonging survival. However, this approach is untenable for the health care system and has a 40% failure rate at 2 years and might cause HF requiring heart transplant. During the last two decades, substantial research and progress were made resulting in a shift in focus from disease treatment to disease prevention and etiology-driven personalized approach. This approach has improved substantially the prognosis of DCM (1, 7, 8).

This review will briefly address the diagnosis, etiologies, and pharmacological and non-pharmacological management of DCM. The main focus will go to the emerging potential immunomodulator agents being developed to treat DCM. The current and future clinical practice through guided individualized treatment strategies bring promises to improve the patients' outcomes and reduce treatment costs.

## Diagnosis

The signs and symptoms of DCM may be fulminant, acute, subacute or chronic, as they are related to the extent of left ventricular or biventricular systolic dysfunction. The diagnosis of DCM is evident by chamber dilation and reduced systolic function of one or both ventricles with an ejection fraction <50%. A thorough evaluation and wide array of non-invasive and invasive techniques are needed to ascertain DCM diagnosis. Non-invasive imaging techniques like electrocardiogram, echocardiogram, chest X-ray screens and cardiac magnetic resonance (CMR) imaging are used to detect and assess enlarged cardiac silhouette, chamber size dimensions, ventricular dysfunction, strain abnormalities, contrast enhancement, the presence of oedema and/or fibrosis, abnormalities ranging from isolated T-wave changes to adverse myocardial remodeling as featured in DCM cases (9, 10). Contrast agents, mainly gadolinium, have been used to more efficiently evaluate fibrosis and subsequently the information is being used as a predictor of future hospitalization and all-cause mortality. Invasive methods such as coronary catheterization is used to rule out any other coronary artery diseases.

In patients with idiopathic DCM, genetic/familial reasons should be considered. Familial DCM diagnosis is ascertained when the proband has two or more first-degree relatives who experienced premature sudden cardiac death aged <35 or heart failure without a definitive cause or by three-generation history of DCM (1). Thus, genetic testing and sequencing the entire coding region of the gene in particular is a hallmark to identify the disease-causing mutations, along with detailed family history (10, 11). Such techniques would help to determine the disease cause and have a tailored risk stratification for etiology-driven therapeutic options for patients.

Additional considerations should however be added to other subcategories of DCM, mainly inflammatory cardiomyopathy. Inflammatory DCM (DCMi) or inflammation of the myocardium is best regarded as any heightened humoral or cellular immune response in the heart with various symptoms such as chest pain, mild dyspnea, or acute cardiogenic shock (12, 13). For definitive diagnosis of myocarditis (myocardium inflammation) and inflammatory DCM (DCMi), Endo-Myocardial-Biopsy (EMB) is used because it detects viral and non-viral causes in the acute and chronic stage of the disease.

Before highlighting standard and personalized approaches to treat DCM (sections Targeted Treatments for DCM and Emerging Immunomodulator Therapeutic Strategies), we will first provide an overall picture on the environmental and genetic etiologies of the disease (sections Environmental and Genetic Etiologies of DCM and Treatment With Conventional Medications).

## Environmental And Genetic Etiologies Of Dcm

### Environmental Etiologies

The cellular changes that result in DCM begin first as an adaptational response but are then transformed into a detrimental cellular, and organ “malaise” as a consequence of accumulated uncontrolled molecular events. We hereby refer to the microenvironment as the environment that surrounds the myocyte. This includes the surrounding cells and their secreted proteins and growth factors in the myocardial interstitium: endocardial, fibroblast, blood cells. In DCM, the myocardial interstitium is constantly subjected to an increase in the extracellular matrix content and reduction in collagen linkage, resulting in faulty matrix and Left Ventricular (LV) dilation (14). Studies have shown that any increase in the production of matrix metalloproteinases (MMPs), or Galectin-3, can be sensed as an early marker for DCM (15). In parallel, cytokines secreted from inflammatory cells and/or oxidative stressors that cause an increase in oxygen reaction species production could directly affect myocardial function and subsequently lead to DCM. As such, fibrosis is a common feature for both genetic and non-genetic dilated cardiomyopathies and constitutes a converging focal point to develop novel drugs to stop the progression of the disease.

In contrast, the macro-environment refers to the overall body adaptation/response to extra-cardiac “insults” and is sensed through a hemodynamic and/or hormonal overload on the heart (14). Examples of such conditions could be obesity, diabetes, infection, drug intolerance, toxicity, viruses, and autoimmune diseases. Table 1 summarizes some of the etiologies of DCM, along with the tailored-diagnostic approaches and specific treatment options, and the following section will highlight some of the direct and indirect “environmental stressors” that leads to DCM.

**Table 1:** Etiologies, diagnosis, and targeted treatments for DCM.

**Etiologies**	**Diagnosis**	**Targeted treatments**
Genes: titin, myosin 7/β-myosin heavy chain, cardiac muscle troponin T, RNA-binding motif protein 20	Family history, Gene testing, whole exome sequencing	If LVEF of <35%, use implantable cardioverter-defibrillators (ICDs).
Toxins: alcohol, cocaine, and cytostatics	Urine test, patient history, family interview, abuse history	Avoid exposure to toxins, pharmacological management for abstinence along with standard therapy
Cardiotropic viruses: like parvovirus B19 (B19V), human herpes virus-6 (HHV-6), and enterovirus Coxsackie B (CVB3)	Endo Myocardial Biopsy (EMB)	Antiviral therapies: IVIG administration, Betaferon, Ganciclovir, and Telbivudine
Medications - Antineoplastic drugs like Anthracyclines (Doxorubicin, Daunorubicin), alkylating agents (Cyclophosphamide), antimicrotubular molecules (Paclitaxel, docetaxel and Vinca alkaloids) and antimetabolites (Capecitabine, Cytarabine, 5-Flourouracil).	Based on prescription and patient history	Avoid usage or using alternative medications with same mechanism of action
Auto immune diseases: systemic lupus erythematosus, systemic sclerosis, rheumatoid arthritis, Kawasaki disease–related myocarditis, Lupus erythematosus, Cardiac sarcoidosis, and giant cell myocarditis	Endo Myocardial Biopsy (EMB), cardiac imaging, blood tests	Immunoadsorption therapy
Endocrine or metabolic diseases: diabetes	Blood tests-Hbalc, glucose tolerance test, cardiac tests and imaging, symptoms	Glycemic control, medications, diet modification, diabetology consultation
Nutritional deficiency in: carnitine, thiamine and selenium	Blood tests and detailed examination	Follow balanced diet

#### Diabetes

Diabetes is a known risk factor for cardiovascular diseases (16, 17). Development of diabetes causes systolic and diastolic dysfunction, thus leading to dilated cardiomyopathy. This development is related to insulin resistance, metabolism of fatty acids, hyperglycemia, and excessive activation of renin angiotensin system. Hyperglycemia, the main driving force of diabetic cardiomyopathy triggers various responses leading to heart failure. Glucose uptake causes increased oxidation and lipotoxicity of the myocardium as well as insulin resistance leading to the initiation of the renin angiotensin aldosterone system. This results in fibrosis and hypertrophy causing myocardial oxygen demand and alteration of calcium storage in the sarcoplasmic reticulum. These series of events eventually lead to a decrease in cardiac contractility, thus causing DCM (18, 19). Recent studies also showed that DCM associated with diabetes could result from the T helper (Th)-driven inflammatory functional and biomolecular changes bestowed on the cardiomyocytes (17). Moreover, a study on 206 DCM patients showed that the disease prognosis in DCM patients with type 2 diabetes is worse than patients without diabetes, in which 15 deaths, 43 hospitalizations and a new onset of atrial fibrillation were reported (19).

#### Autoimmunity

DCM can result from autoimmune diseases such as systemic lupus erythematosus, systemic sclerosis, rheumatoid arthritis, Kawasaki disease–related myocarditis, lupus erythematosus, cardiac sarcoidosis, and giant cell myocarditis (20, 21). In patients with DCM of autoimmune etiologies, B cells produce cardiac-specific autoantibodies (AABs) such as the ones targeting the β1-adrenergic receptor, the muscarinic M2-acetylcholine receptor, the Na-K-ATPase pump, and Troponin I. These AABs form immune complexes with self-antigens and complement components. AABs influence myocytes function directly as pathogenic agents secondary to tissue aggression and are known to be present in 60% of patients with DCM (22, 23).

#### Toxic Environment

Some of the toxins that can cause DCM are alcohol, cocaine, and cytostatics (10). Left ventricular dysfunction and DCM have been related to heavy drinking with increased rate of cardiac morbidity and mortality. In regards to cocaine, it was shown that high cocaine doses cause reduction in left ventricular ejection fraction (LVEF) leading to dilated cardiomyopathy (24).

#### Drugs

Several anti-neoplastic drugs are cardiotoxic like Anthracyclines (Doxorubicin, Daunorubicin), alkylating agents (Cyclophosphamide), antimicrotubular molecules (Paclitaxel, docetaxel and Vinca alkaloids) and antimetabolites (Capecitabine, Cytarabine, 5-Flourouracil). The function, and metabolic activity of the heart are drastically perturbated by these agents (25, 26). Doxorubicin causes cardiotoxicity by generating oxygen reactive species that affect the whole contractile machinery leading to Doxorubicin-induced cardiomyopathy (DiCM). Antiretroviral agents like azidothymidine have cardiotoxic properties, as in the case of Doxorubicin, through increased generation of reactive oxygen species. Finally, immune check point (ICI) therapies like Programmed Death-Ligand 1/2 (PDL1 and PDL2) inhibitors have been successful in the improvement of advanced cancer stages. However, recent studies have shown they cause cardiac toxicity, myocarditis, decreased LV function, and immune related adverse events (27).

#### Viruses

Cardiotropic viral infections induce cardiac dysfunction and may lead to DCM. The predominant viral cause seems to change with every decade (coxsackievirus in the 1980s, adenovirus in the 1990's, and parvovirus B19 since 2000) (9). In developed countries, adenoviruses and enteroviruses were mostly recognized until the 1990's. However, in recent years cardiotropic viruses like, parvovirus B19 (B19V), human herpes virus-6 (HHV-6), and enterovirus coxsackie B (CVB3) are significantly increasing in the population with cardiomyopathy. Overall, enteroviral genomes were found in 3–53%, cytomegalovirus in 3–40%, and adenoviruses in 3–23% of the myocardium (28). In viral infection, overexpression of the inflammatory cytokines like Tumor Necrosis Factor alpha (TNFα) causes initiation of the immune system response cascade that directly affects the function of cardiomyocytes and their survival. Infiltrating immune cells have a key role in eliminating infected myocardial cells and limiting viral replications in the heart but as such they contribute to the worsening of the phenotype by eliminating cardiomyocytes through apoptosis. Cytotoxic T lymphocytes (CTLs) are responsible for lysing virus-infected cardiomyocytes which leads to further myocardial cell damage. They recognize virus-derived peptides presented in the groove of the major histocompatibility complex (MHC) molecule class 1 antigen through T-cell receptors and play a key role in the pathogenic immune mechanism in viral myocarditis and DCM (29). Overall, the viruses might cause direct myocardial damage or a secondary virus-initiated myocardial injury where the end-results combine a series of molecular and cellular events like myocyte necrosis and fibrosis that lead to DCM (30).

#### Inflammatory Response: Cytokine Storm

As discussed above, TNF-α overexpression causes a series of cellular events that causes cardiomyocyte inflammation/necrosis. TNF-α and other cytokines such as interleukin-1 (IL-1), interleukin-6 level (IL-6), interleukin-10 (IL-10), and interleukin-18 (IL-18) are an essential part of the inflammatory process. Cytokines are produced by several immune cells like the innate macrophages, dendritic cells, natural killer cells and the adaptive T and B lymphocytes. Accumulating evidence suggest that patients with DCM suffer from a “cytokine storm.” The “cytokine storm” results from a sudden acute overexpression in circulating levels of IL-6, IL-1, IL-10, IL-18, TNF- α, and interferon. This cytokines overexpression results in influx of various immune cells such as macrophages, neutrophils, and T cells from the circulation into the site of infection with destructive effects in cardiovascular cells resulting to capillary damage and myocardial fibrosis leading to DCM (31–33). Further studies are needed to establish the mechanisms of the cytokine storm/dysregulation concretely, which can explain the pathogenesis/prognosis of DCM.

### Genetic Etiologies of DCM

A spectrum of genetic heterogeneity underlies DCM and accounts for half of the cases. At current, a list of 42 genes is used worldwide as a blueprint for all cases of cardiomyopathies including DCM. Most of these genes encode proteins implicated in the structure of the muscle heart cells like the sarcomeres, the Z-disks, and sarcolemma; consequently, alterations in their structure and/or function would affect muscle contraction. Examples of these genes are *LMNA* or *SCN5A, BAG3, FLNC, PLN, RBM20*, and *TTN*. Identifying the heredity can be difficult due to the incomplete penetrance and variable expressivity of DCM and genetic variation. Thirty-five percent of DCM cases have a family history, inherited in Autosomal Dominant manner (AD), in some cases autosomal recessive or X-linked inheritance traits (3, 34). We will cover herein the known genetic variants in genes that encode for sarcomeric, Z-disc and laminal membrane proteins.

#### Genes Encoding Sarcomeric Proteins-TTN (Titin) Mutations

*TTN* encodes Titin, one of the largest proteins in humans and harbors the most frequent genetic variants associated with DCM. The protein plays a structural role in maintaining the thick filaments stability within the sarcomere by avoiding the filaments overstretching. *TTN* genetic variants show a consistent high penetrance trait for familial DCM cases. Due to the enormous size of the gene along with the frequency of *TTN* variants in the reference population, it is challenging to interpret the variant as pathogenic, pathogenic, singleton or familial (35). The most frequent variant is a founder mutation leading to truncation of the C-terminal part of the protein (36). The proteotoxic effect of such misfolded and/or non-functional aggregates of TTN proteins in cardiomyocytes is the direct cause of DCM (37). The clinical phenotype of *TTN* mutations involves a tendency for left ventricular remodeling and dysfunction, atrial fibrillation, frequent ventricular ectopy, and non-sustained ventricular tachycardia, and malignant ventricular arrhythmia (38, 39).

Moreover, reduced expression of titin is believed to be associated with the pathophysiology of DCM. A significant decrease in titin and dystrophin mRNA and protein levels was seen in endomyocardial biopsy of DCM patients as compared to control, the severity of the disease correlated with this decrease.

The study suggested that TNF-α might modulated the expression of titin and dystrophin levels via nuclear factor kappa B (NF-kappaB) pathway. To confirm that, TNF-α was used as a treatment and resulted in a dose- and time-dependent decrease in mRNA levels of dystrophin and titin (40). Other studies supported this hypothesis, where they revealed that TNF-α gene polymorphism (G-308A) might play a key role in the susceptibility to dilated cardiomyopathy (41).

#### Genes Encoding Nuclear Laminal Proteins-LMNA Mutations

L*MNA* encodes Lamin A/C, an envelope protein that acts as a support element to intermediate filaments and regulates gene expression by stabilizing the inner nuclear membrane (42). After *TTN* variants, *LMNA* are the second most common DCM-causative mutations. Mutations in *LMNA* are inherited in AD manner and might be associated with other autosomal dominant disorders such as Emery-Dreifuss muscular dystrophy. *LMNA* variants increase the risk of sudden cardiac death up to 46%, cardiac muscular atrophy, premature aging, systolic dysfunction and high prevalence of arrhythmias, disturbance of signal transduction in non-dividing cells and disturbance of chromatin organization in dividing cells (38, 43, 44). Pathogenesis of LMNA-associated DCM includes disturbance of signal transduction in non-dividing cells and disturbance of chromatin organization in dividing cells. The common features associated with *LMNA* mutations in DCM patients are the coexistence of a defect in mechano-transduction and laminopathy development with conduction system abnormalities resulting in diverse phenotypes. Phenotypes such as lipodystrophy, skeletal and/or cardiac muscular atrophy, dysplasia, premature aging, systolic dysfunction and high prevalence arrhythmias and other neuromuscular diseases which result in poor prognosis and response to medical treatment (38, 42).

### Genes Encoding RNA Binding Proteins-RBM20 Mutations

Mutations in the gene encoding the RNA-binding motif 20 (RBM20), a nuclear phosphoprotein mainly expressed in the cardiac myocytes have been emerging as one of the latest causes of familial DCM cases despite being first linked to arrhythmogenic cardiomyopathies (45, 46). The link to the DCM phenotype has been recently explored, and as such the role of RBM20 as a master regulator of alternative splicing of genes involved in the contractile machinery namely Titin and Lamin has been pointed out (47, 48).

With all the etiologies being exposed, the following sections will first provide a current summary of the ongoing and proposed clinical trials that use conventional treatment and etiology-driven therapeutic treatments.

## Treatment With Conventional Medications

Conventional medications are the first line drug treatment that have been studied in large clinical scale trails and shown survival improvement and reduction in hospital admission. Conventional treatment is based on the classification of the patients as per “measured” clinical criteria. The New York Heart Association (NYHA) classified DCM patients into five groups based on their heart failure. Class I: patients with cardiac disease but without resulting limitations of physical activity, and ordinary physical activity does not cause undue fatigue, palpitation, dyspnea, or anginal pain. Class II: patients with cardiac disease resulting in slight limitation of physical activity, are comfortable at rest and ordinary physical activity results in fatigue, palpitation, dyspnea, or anginal pain. Class III: patients with cardiac disease resulting in marked limitation of physical activity, are comfortable at rest, and less-than- ordinary physical activity causes fatigue, palpitation, dyspnea, or anginal pain. Class IV: patients with cardiac disease resulting in inability to endure physical activity without discomfort. Symptoms of cardiac insufficiency or of the anginal syndrome may be present even at rest. If any physical is undertaken, discomfort is increased. Overall, the treatment of each class varies between the use of Angiotensin Converting Enzyme Inhibitors (ACEIs) (49–51), Angiotensin Receptor Antagonists Losartan (52, 53), β blockers (54–57), Aldosterone antagonists (58, 59), Ivabradine (60), Angiotensin receptor-neprilysin inhibitors (61). Table 2 summarizes the pharmacological management of DCM based on NYHA and the American Heart Association (AHA) class of recommendation (63, 72).

**Table 2:** Pharmacological treatment of DCM.

**References**	**(n)Targeted population**	**Study design**	**Drug**	**Assessment of outcome**	**Outcome**	**Class of recommendation by AHA**
**Conventional drugs**						
**ACEIs**						
Group (49)	(253) NYHA IV; 38 (15%) with non-ischaemic cardiomyopathy	Double-blind, placebo-controlled, parallel group	Enalapril (120 mg twice a day)	1 year	Improved survival and NYHA class; mean dose 18.4 mg/day	I
Investigators et al. (50)	(2569) NYHA II–III; 469 (18%) with non-ischaemic cardiomyopathy	Double-blind, placebo-controlled, parallel group	Enalapril (10 mg twice a day)	3 years	Improved survival and fewer hospital admissions; mean dose 11 mg/day	I
Investigators et al. (51)	(4228) NYHA I; 396 (10%) with non-ischaemic cardiomyopathy	Double-blind, placebo-controlled, parallel group	Enalapril (10 mg twice a day)	3 years	Improved survival, fewer hospital admissions, and less HF progression; mean dose 12.7 mg/day	I
**Angiotensin receptor antagonists**						
Pitt et al. (52)	(3152) NYHA II-IV; ≥60 years; 1,292 (41%) with non-ischaemic cardiomyopathy	Double-blind, active-controlled	Losartan (50 mg once a day) vs. captopril (50 mg three times a day)	1.5 years	There were no significant differences in all-cause mortality	I
Granger et al. (53)	(2028) NYHA II-IV; β blocker; 396 (20%) with non-ischaemic cardiomyopathy; angiotensin converting enzyme inhibitor intolerant	Double-blind, placebo-controlled	Candesartan Cilexetil (32 mg once a day)	2 years	Reduced cardiovascular mortality and morbidity in patients with symptomatic chronic heart failure	I
**β blockers**						
Packer et al. (54)	(1094) NYHA II-IV; 570 (52%) with non-ischaemic cardiomyopathy; angiotensin converting enzyme inhibitor or angiotensin receptor blocker	Double-blind, placebo-controlled	Carvedilol (various dosing)	6–12 months	Mortality rate was 7.8 percent in the placebo group and 3.2 percent in the carvedilol group; the reduction in risk attributable to carvedilol was 65 percent (95% confidence interval, 39–80%; *P* < 0.001)	I
(55)	(2674) NYHA class III-IV; 317 (12%) with non-ischaemic cardiomyopathy; angiotensin converting enzyme inhibitor or angiotensin receptor blocker	Double-blind, placebo-controlled	Bisoprolol (10 mg once a day)	1.3 years	All-cause mortality was significantly lower with bisoprolol than on placebo (156 [11.8%] vs. 228 [17.3%] deaths)	I
(56)	(3991) NYHA II-IV; 1,385 (35%) with non-ischaemic cardiomyopathy; angiotensin converting enzyme inhibitor or angiotensin receptor blocker	Double-blind, placebo-controlled	Metoprolol controlled release (200 mg once a day)	1 year	The total mortality or all-cause hospitalizations was lower in the metoprolol CR/XL group than in the placebo group (641 vs. 767 events; risk reduction, 19%; 95% confidence interval [CI], 10–27%; *P* < 0.001)	I
Packer et al. (57)	(2289) NYHA III-IV; 755 (33%) with non-ischaemic cardiomyopathy; angiotensin converting enzyme inhibitor or angiotensin receptor blocker	Double-blind, placebo-controlled	Carvedilol (25 mg twice a day)	4 years	Carvedilol reduced the risk of death by 39 percent (95 percent confidence interval, 11–59 percent; *P* = 0.009) and decreased the combined risk of death or hospitalization by 29 percent (95 percent confidence interval, 11–44 percent; *P* = 0.003).	I
**Aldosterone antagonists**						
Pitt et al. (58)	(1663) NYHA III-IV; 765 (46%) with non-ischaemic cardiomyopathy; angiotensin converting enzyme inhibitor or angiotensin receptor blocker	Double-blind, placebo-controlled	Spironolactone (25 mg once a day)	2 years	Reduced mortality and hospital admissions	I
Zannad et al. (59)	(2737) NYHA II; 846 (31%) with non-ischaemic cardiomyopathy; angiotensin converting enzyme inhibitor or angiotensin receptor blocker + β blocker	Double-blind, placebo-controlled	Eplerenone (25–50 mg once a day)	21 months	Reduced mortality;18.3% of patients in the eplerenone group as compared with 25.9% in the placebo group (hazard ratio, 0.63; 95% confidence interval [CI], 0.54–0.74; *P* < 0.001)	I
**Ivabradine**						
Swedberg et al. (60)	(6558) NYHA II-IV; sinus rhythm with heart rate of >70 beats per min; 2,087 (33%) with non-ischaemic cardiomyopathy; angiotensin converting enzyme inhibitor or angiotensin receptor blocker + β blocker	Double-blind, placebo-controlled	Ivabradine (5–7.5 mg twice a day)	18–28 months	24% patients in the ivabradine group had cardiovascular death or hospital admissionand, compared to 937 (29%) of those taking placebo (HR 0.82, 95% CI 0.75–0.90, *p* < 0.0001)	IIa
**Angiotensin receptor-neprilysin inhibitors**						
McMurray et al. (61)	(8442) NYHA II-IV; 3,363 (40%) with non-ischaemic cardiomyopathy; angiotensin converting enzyme inhibitor or angiotensin receptor blocker; β blocker	Double-blind, active-controlled with enalapril	Sacubritil valsartan (200 mg twice a day)	27 months	As compared with enalapril, sacubritil valsartan reduced the risk of hospitalization for heart failure by 21% (*P* < 0.001) and decreased the symptoms and physical limitations of heart failure (*P* = 0.001)	I
**Targeted therapy for viral causes**						
McNamara, et al. (62)	(63) Patients with recent onset (< /= 6 months of symptoms) of dilated cardiomyopathy and LVEF < / = 0.40	Prospective randomized, placebo-controlled, double-blind	Intravenous immunoglobulin(IVIG2) 2 g/kg	2 years	LVEF improved from 0.25+/−0.08 to 0.41+/−0.17 at 6 months (*P* < 0.001) and 0.42+/−0.14 (*P* < 0.001 vs. baseline) at 12 months	
Dennert et al. (64)	(17)Patient with Parvovirus B19 DCM	Uncontrolled pilot study	(IVIg) (2 g/kg) for 6 months	9 months	Decrease in EMB viral load (*P* = 0.004) and improved LVEF (*P* = 0.001).	
Zimmermann et al. (65)	(110) patients with chronic viral DCM	Open trial with untreated control group	Interferon β-1b for 6 months	3 years	No benefit of interferon β-1B treatment observed.	
Schultheiss et al. (66)	(143) patients with symptoms of heart failure and biopsy-based confirmation of the enterovirus (EV), adenovirus, and/or parvovirus B19 genomes)	Double-blind treatment	Interferon β-1b for 6 months	1 year	Improvement in quality of life	
**TARGETED THERAPY FOR NON-VIRAL (INFLAMMATORY) CAUSES**						
**Immunosuppresive drugs**						
Parrillo et al. (67)	(102) patients with Idiopathic Cardiomyopathy	Prospective, randomized, controlled	Predinsone (60 mg a day)	3 months	Marginal clinical benefit, and should not be administered as standard therapy for dilated cardiomyopathy.	
Wojnicz et al. (68)	(67) DCM patient with increased HLA expression	Randomized, placebo-controlled	Prednisone + azathioprine (3 months)	2 years	At the end of the follow-up period, 71.4% patients from the immunosuppression group vs. 30.8% patients from the placebo group were improved (*P* = 0.001).	
Frustaci et al. (69)	(70) Myocarditis and chronic (>6 months) heart failure patients, unresponsive to conventional therapy, with no evidence of myocardial viral genomes	Randomized, double-blind, placebo-controlled	Prednisone + azathioprine (6months)		improvement of LV-EF and decrease in LV dimensions and volumes compared with baseline with no major adverse reaction	
Escher et al. (71)	(114) Chronic myocarditis or inflammatory cardiomyopathy following Caforio et al. (≥14 infiltrating inflammatory cells/mm^2^)	Retrospective case series	Immunosuppresion (6 months)	3 years	Improvement of LV-EF compared to baseline after 6-mo period (LV-EF rising from 44.6 ± 17.3% to 51.8 ± 15.5%; *P* = 0.006)	
Merken et al. (70)	(209) Inflammatory cardiomyopathy following Caforio et al. (≥14 infiltrating inflammatory cells/mm^2^)	Retrospective case series	Immunosuppresion (1 year)	≤ 10 years	A significant larger increase of LV-EFmproved long-term outcome (e.g., heart transplantation-free survival)	

## Targeted Treatments For Dcm

In general, with conventional therapies, ~25% of DCM patients with recent onset symptoms of HF will have spontaneous improvement, but patients with symptoms lasting >3 months who show severe clinical presentations generally have less chance of recovery, thus treatments will be recommended to optimize heart function, reduce the risk of worsening disease, prevent complications, and/or reduce symptoms caused by heart failure. Non-pharmacological treatment is strongly recommended for etiologies that can be controlled such as avoiding exposure to toxins (e.g., alcohol, cocaine). In alcoholic cardiomyopathy (aCM), abstinence from alcohol has shown to improve the LVEF in patients. A study conducted for 82 months on 101 aCM patients showed a noticeable improvement in LVEF function (i.e., QRS duration <120 ms) for 42% of them (73). Also, following a balanced healthy cardiac die or treating endocrine disorders (e.g., diabetes, thyroid disease) should be considered (6). Patients with DCM and diabetes should be educated on glycemic control and the importance of adhering to medication to order to avoid complications (74). For patients with genetic causes, the clinical management is based on the clinical features associated with the genetic information. The first approach is to improve the clinical outcomes based on a definite genetic mutation. The primary preventive method for patients with genetic background with LVEF of <35% is to use implantable cardioverter-defibrillators (ICDs) to reduce sudden cardiac death (SCD). To reduce the risk of instabilities in cardiac rhythm and SCD in DCM patients with LMNA mutation, the threshold frequency for implanted cardiac defibrillators should be lowered (75). Bi-ventricular pacing is recommended for symptomatic bradycardia that show left bundle block in DCM patients. Studies on DCM patients undergoing cardiac resynchronization therapy showed improved survival, quality of life along with reduced hospital admissions (76).

### Targeted Therapy for Genetic Causes

#### Known Drugs

The second approach is targeting the defect gene mutations at the molecular level directly by affecting the structure and function of the encoded proteins. Currently, an allosteric modulator was developed to directly bind in the same region on myosin, increasing actin affinity and cross-bridge formation, as well as enhancing sarcomere force production. This modulator is well-studied in patient with reduced LVEF from heart failure, which can benefit DCM patients since they have reduced LVEF. CK-1827452 also known as Omecamtiv mecarbil (INN) accelerates the transition of actin-myosin complex from weakly to strongly bound and increases the number of myosin heads engaged with the thin filament. These effects are independent of calcium transients because CK-1827452-treated cardiomyocytes with isoproterenol augment contraction, whereas β-adrenergic inhibition does not diminish contractility (77) ATOMIC (78). Additional studies indicated that CK-1827452 traps some myosin heads in a weak actin affinity state with slow force development and at high concentrations prolonged cellular relaxation in hiPSC-CMs (79). A randomized, parallel-group, double-blind, phase II conducted over 87 sites in 13 countries (The Chronic Oral Study of Myosin Activation to Increase Contractility in Heart Failure COSMIC-HF trial) showed that CK-1827452 administered to patients with chronic stable symptomatic heart failure increased stroke volume and modestly reduced left ventricular end-diastolic diameter, heart rate, and serum levels of N-terminal brain natriuretic factor, increased systolic ejection time, and it may have improved dyspnea in the high-dose group, though it did not meet the primary endpoint of dyspnea improvement. Many other trails have been conducted and are still under investigation to study this drug (Table 2) (77, 78, 80).

#### Novel Drugs

Specific inhibitors to the pathogenic effects bestowed by the pathogenic mutations are postulated to be beneficial in treating DCM. Pre-clinical trials using novel small molecules did yield encouraging results. The LMNA mouse model was treated with ARRY-371797, an oral medication that inhibits the p38 MAP kinase: LV dilation was prevented, and EF substantially improved. To investigate the benefits of ARRY-371797 in DCM patients with LMNA mutations, patients with LMNA mutations, a clinical trial is currently ongoing to evaluate its effectiveness based on changes in the 6 min-walk tests over a 24 weeks-time period (NCT03439514). With the increasing focus on the role of RBM20 as a master regulator of alternative splicing, recent data suggest that in patients with reduced RBM20 activity, all-trans retinoic acid (ATRA) could be used to restore RBM20 levels and efficiently curb down the deleterious effects of loss of function mutations in this gene (81).

A novel myosin activator is Danicamtiv (formerly known as MYK-491). It is a new targeted myosin activator under development for the treatment of DCM. It accelerates and activate myosin contractility directly by increasing cross-bridge formation with no effects on the calcium transient. This has the potential to improve the hemodynamic profile of patients with systolic heart failure while avoiding the energetic consequences of adrenergic agonists and phosphodiesterase inhibitors. It was characterized in *in vitro* and *in vivo* (in mice, rats, dogs, and monkeys) preclinical studies and this led to the support of its advancement into clinical investigations (82).

### Targeted Therapy for Viral Causes

Antiviral therapy treatment is suggested for viral causes of DCM and currently there are many trials ongoing to understand their beneficial effects at distinct phases. In summary, patients with enterovirus are treated with Interferon beta, Parvovirus B19 with immunoglobulins and Telbivudine and Human Herpesvirus Type 6-Positive Patients with Ganciclovir and this will be further explained below in details (Table 2) (83).

#### Immunoglobulin

Immunoglobulins reduces oxidative stress by preventing the development of proinflammatory cytokines; thus, they have both antiviral and anti-inflammatory properties (84). Intravenous Immunoglobulin (IVIG) treatment for DCM patients has been controversial Improved LVEF function with reduced amount of virus load and lower hospitalization rates was noted in DCM patients receiving IVIG (64). Studies have shown that children and infants with DCM infected by human parechoviruses benefited from IVIG therapy (85, 86). DCMi patients treated with Ig-Therasorb and immunoglobulin G (IgG) showed a continued improvement in the cardiac index (87). In patients with virus positive, the therapeutic dosage for treatment with IVIG (IgG, IgA, and IgM- Pentaglobin) is 10–15 g (variable according to weight) on day 1 and day 3. High doses of intravenous 1 g of dose (2.0 g/kg body weight) were administered for patients with B19v for viral load reduction. There was no improvement in cardiac function and quality of life after this 4-day treatment. This negative finding could be related to the number of days of treatment with IVIG in this patient population (88). This finding also emphasizes on the fact that multifactorial causes of DCM make it difficult to have a unidirectional treatment.

#### Telbivudine

Telbivudine, an analog of thymidine is an antiviral nucleoside that has immunomodulatory-antiapoptotic properties. The PreTopic Study assessed the effect of Telbivudine on B19V-positive patients (83). Patients with less B19V DNA load having inflammation of the cardiac cells have benefited from the drug while those with high viral loads did not show significant improvement (89). Telbivudine inhibited viral replication, and reduced inflammation while improving cardiac function. In addition, Telbivudine did reduce cardiomyocyte apoptosis (71, 83); however, there are no randomized clinical studies to evaluate the results of Telbivudine, and as such it cannot be used as a standard treatment.

#### Interferon Beta (IFN-ß)

Anti-viral treatment with type I interferons was shown to be a suitable choice for viral positive cardiomyopathies. A non-randomized study showed administration of INF-ß results in improved survival by reducing the virus induced injury to the myocardium. The study showed that patients who received IFN-ß had higher virus elimination rates, improved quality of life and fewer adverse cardiac associated occurrences (66).

### Targeted Therapy for Inflammatory Causes-Immunosuppressants

Treatment guidelines specific to inflammatory causes of DCM are scare. Extensive multicenter studies need to be conducted to prove the effectiveness of the treatment options (28). Current studies suggest that treatment need to be initiated at the early phase before the symptoms becomes worse and chronic (12). Early studies (1980's and 1990's) concluded that the use of immunosuppressive like prednisone, cyclosporine and azathioprine has a minimal clinical benefit and should not be used as standard therapy for DCM. The improvement was observed at the beginning of the trails, but it was not constant on extended period of time and there was no differences in the disease progression upon using these immunosuppressant agents (67). This might be due to the limitation in diagnosis and initiating this therapy to patient with virus positive DCM, thus determining the etiology of DCMi is important before initiating an immunosuppressive treatment regimen. There is no evidence of a guideline directed therapy but there are recent studies showing treatment with Immunosuppressants to beneficial. Studies showed the higher efficacy of immunosuppressive therapy when combined with regular heart failure medications in patients with biopsy-proven, virus-negative inflammatory cardiomyopathy when initiated before irreversible remodeling occurs (70, 71) (Table 2). In virus negative patients with inflammatory DCM, prednisolone and azathioprine was given for 3 months and were evaluated for 2 years in a randomized placebo-controlled study. Results showed that 71.4% patients from the immunosuppression group vs. 30.8% patients from the placebo group were improved (*P* = 0.001) (68). A larger retrospective case series from the Innsbruck and Maastricht Cardiomyopathy Registry showed that immunosuppressive therapy (azathioprine, prednisone, and cyclosporine) is associated with heart transplantation-free survival as compared with standard heart failure therapy alone (70). Other retrospective analysis showed that immunosuppressive treatment of patients with virus-negative inflammatory cardiomyopathy showed an improvement of LVEF with no major adverse reaction (69).

### Targeted Therapy for Autoimmunity-Immunoadsorption

The assumption that disease associated auto antibodies (AAB) play a role in DCM pathogenesis, entail that removing such antibodies will result in improving the patients' cardiac parameters. Few studies have been conducted and some are still underway. There was an improvement in the pulmonary capillary output pressure, cardiac index, pulmonary resistance, reduction in AAB levels when immunoadsorption therapy was done for 5–7 cycles on 9 patients with DCM and increased anti-beta adrenergic receptor AAB levels (84). The results of other studies that followed up with patients for 3 months and 1 year showed significant improvement in the LVEF function (69.9%) (87). Patients who can benefit from this intervention can be identified using a combination of assessment of negative inotropic activity of antibodies and expression of gene patterns (90). In a study 4 courses of IA therapy administered for 3-month period intervals showed improvement in LVEF along with reduction of LV factor. Recently, Aptamer BC 007, a 15-mer single-strand DNA oligonucleotide drug (5′GGTTGGTGTGGTTGG-3′), was developed to neutralize AAB that bind to the extracellular domains of G protein-coupled receptors (GPCR-neutralization), amongst which beta 1 adrenergic receptors. Aptamer BC 007 was shown to improve cardiac function and prolong survival of Doberman Pinschers (DP) with DCM. These results promise that aptamer BC 007 might be effective in human patients with DCM (91). S100A8/A9 might serve as therapeutic targets in inflammatory cardiomyopathies. IA is still in an experimental period and use of it in clinicals require double blinded large multicenter studies (92).

## Emerging Immunomodulator Therapeutic Strategies

**Figure 1:**
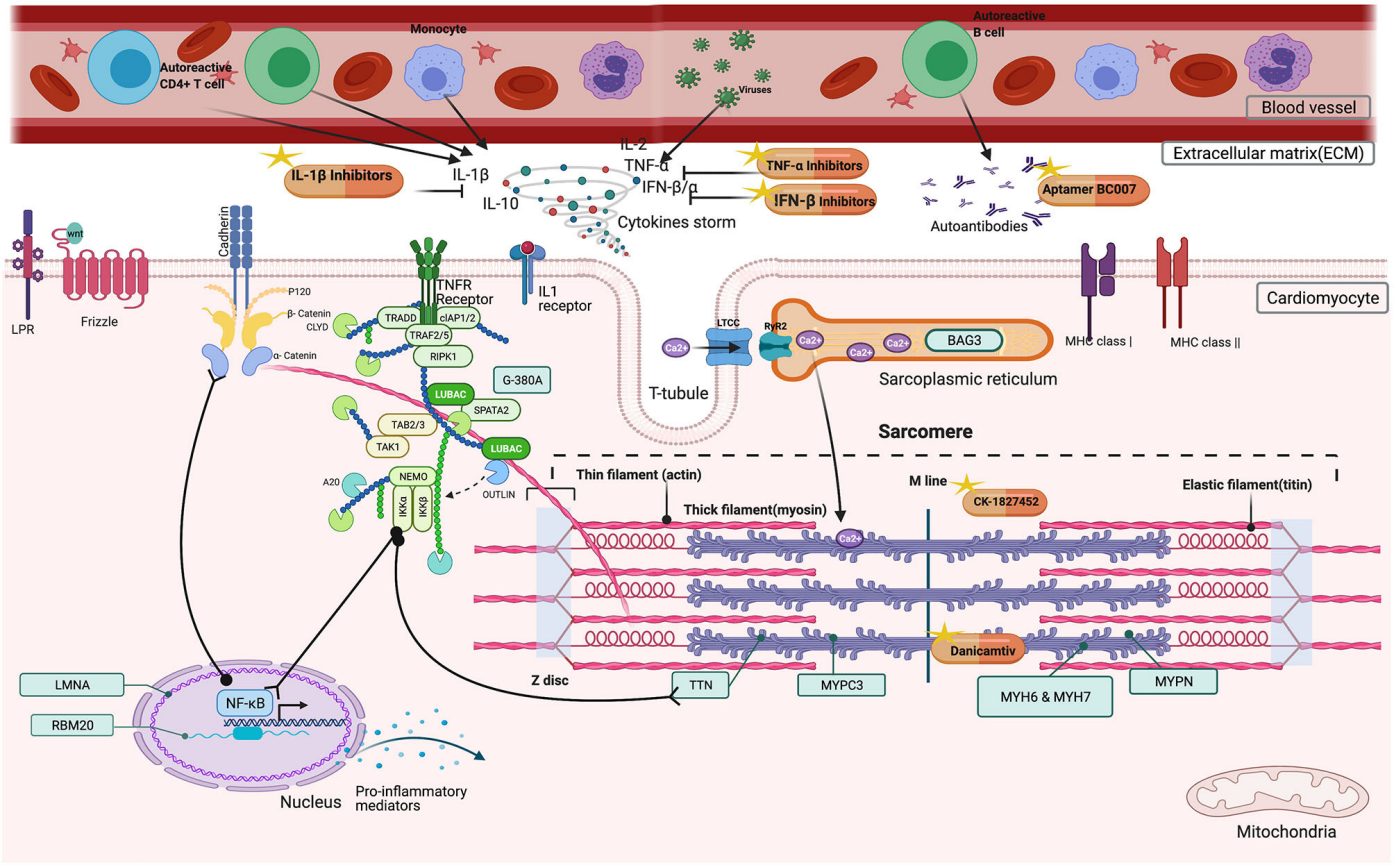
Possible beneficial effects of immunomodulators in DCM patients. Inside the cardiomyocyte, CK-1827452, Danicamtiv accelerates the transition of actin-myosin complex in the sarcomere. In the extracellular matrix (ECM), TNF-α, IL-1β Inhibitors, and IFN β, Interferon beta downregulates the cytokine storm by inhibiting several factors (TNF-α, Tumor Necrosis Factor alpha, IL-1β Inhibitors, Interleukin−1 beta, IL-2, Interleukin-2, IL-10 Interleukin-10, IFN β, Interferon beta, IFN α, Interferon alpha). Aptamer BC007 acts on AAB neutralization.

Substantial emerging anti-inflammatory agents are entering early phase clinical evaluation (Figure 1). These strategies are pathway-specific which include TNF-α inhibitors, IL-1β inhibitors, or immunomodulation by cellular components.

### TNF-α Inhibitors

Up until now, Etanercept and Pentoxifylline are the most studied agents, but there is a lack of recent studies of their effectiveness. The use of Etanercept, a TNF-α antagonist lowered the levels of biologically active TNF in patients with moderate heart failure. The treatment was safe and well-tolerated for 3 months; it led to a significant dose-dependent improvement in left ventricular (LV) ejection fraction and LV remodeling (93). Other studies showed LVEF improvement and a considerable TNF-α production reduction in patients with ischemic cardiomyopathy after receiving pentoxifylline to conventional medications for 6 months (94). These studies have limitations due to small number of patients and short duration of follow-up. More importantly, some contradictions have been raised as to their beneficial effects, and most of the studies have been halted since 2010. Finally, Qiliqiangxin another promising drug that acts on regulating the balance between TNF-alpha and IL-10 showed improvement in cardiac function in rats with myocardial infarction (NCT01293903) (95). Unfortunately, the molecular mechanisms underlying the mode of action of this traditional Chinese medicine were not dissected and studies have been shifted to its usage in heart failure of all causes and not restricted to DCM (96).

### IL-1 Inhibitors

As such, biological treatments that block the IL-1 pathway are potential agents to treat myocarditis such as the IL-Ra (IL-1 receptor antagonist) anakinra, and canakinumab.

#### Interlukin-1a Receptor Antagonists

The IL-Ra antagonist (Anakinra) blocks the acute inflammatory response accompanied with ST-segment elevation in acute myocardial infarction. A study on Anakinra receiving patients showed a lower incidence of heart failure (90). In recently decompensated systolic heart failure, a benefit of prolonged anakinra treatment was suggested by observing improved peak Vo2, with anakinra treatment for 12 weeks, but not for 2 weeks (97). In patients with colchicine resistance and corticosteroid-dependent recurrent pericarditis, over a median of 14 months, anakinra reduced the risk of recurrence (98). In heart failure with preserved ejection fraction, the use of anakinra for 14 days reduced the systemic inflammatory response and improved the aerobic exercise capacity. Conversely, in a group of patients with heart failure with preserved ejection fraction, treatment with anakinra for 12 weeks deteriorated the peak Vo2 (97, 99). Two patients with fulminant myocarditis have recovered after Anakinra was administered along with standard therapy (100, 101). Moreover, the ACTION Study Group in France initiated a Phase 2B double blind randomized controlled trial evaluating ARAMIS (anakinra vs. placebo for the treatment of acute myocarditis) (NCT03018834). The study is estimated to be completed in 2021. Furthermore, Anakinra administration for 4 weeks to a DCM patient showed improvement in LVEF, arrhythmias, ventricular ectopic beat, and myocardial edema. It also resulted in interleukin-6 levels serum reduction, which measures the inflammation induced by interleukin-1 (102).

#### Canakinumab

Canakinumab which is an anti–IL-1β monoclonal antibody has demonstrated reduction of highly sensitive c reactive proteins and 1β, and IL-6 in patients with CAD (CANTOS [Canakinumab Anti-inflammatory Thrombosis Outcome Study] trial). However, its possible benefits in DCM need to be studied further (103).

### Immunomodulation by Cellular Components

Mesenchymal stromal cells cardioprotective and immunomodulatory properties have been well-established and have been shown myocarditis improvement in experimental models of CVB3-induced myocarditis, autoimmune-induced DCMi and chronic Chagas cardiomyopathy (89, 104, 105). In DCM patients, transendocardial injection of autologous and allogeneic mesenchymal stromal cells in non-ischemic have been shown to be safe and clinical efficient in the randomized POSEIDON-DCM trial, through the involvement of the cardiosplenic axis- the homing of immune cells from the spleen to the heart and then subsequent involvement in cardiac remodeling in myocarditis or DCMi (Percutaneous Stem Cell Injection Delivery Effects on Neomyogenesis-DCM) (104).

## Conclusion

There is a necessity to better understand DCM pathogenies and etiologies to tailor the treatment for patients. Many immuno-based therapies are currently available, but refinement of these novel drugs need to be done to better understand the clinical efficacy in patients. Large cohort studies and advanced animal model experimentation need to be carried out prior conducting clinical trials to validate the significance of these etiology-based treatments. A challenge to apply specific tailored therapies would be the stratification of patients based on the causative factors. Effective stratification of patient into virus positive, negative, and inflammatory causes using EMB and imaging methods will allow for causative agent identification that can be used in the diagnosis, prognosis, and initiation of immuno-modulators agents. Thus, more clinical research needs to be conducted to prove the effectiveness of stratification, pharmacological use of immuno-modulators and the significance of it in hospital settings.

## Author Contributions

AK, FM, and GN have made significant contributions to writing this manuscript. All authors contributed to the article and approved the submitted version.

## Conflict of Interest

The authors declare that the research was conducted in the absence of any commercial or financial relationships that could be construed as a potential conflict of interest.
